# Apoptotic HPV Positive Cancer Cells Exhibit Transforming Properties

**DOI:** 10.1371/journal.pone.0036766

**Published:** 2012-05-04

**Authors:** Emilie Gaiffe, Jean-Luc Prétet, Sophie Launay, Elise Jacquin, Maëlle Saunier, Geneviève Hetzel, Pierre Oudet, Christiane Mougin

**Affiliations:** 1 Equipe d'Accueil 3181, Université de Franche-Comté, Besançon, France; 2 Institut Fédératif de Recherche 133, Université de Franche-Comté, Besançon, France; 3 Laboratoire de Biologie Cellulaire et Moléculaire, Centre Hospitalier Universitaire de Besançon, Besançon, France; 4 Hôpitaux Universitaires de Strasbourg, Université de Strasbourg, Strasbourg, France; University of Nebraska Medical Center, United States of America

## Abstract

Previous studies have shown that DNA can be transferred from dying engineered cells to neighboring cells through the phagocytosis of apoptotic bodies, which leads to cellular transformation. Here, we provide evidence of an uptake of apoptotic-derived cervical cancer cells by human mesenchymal cells. Interestingly, HeLa (HPV 18+) or Ca Ski (HPV16+) cells, harboring integrated high-risk HPV DNA but not C-33 A cells (HPV-), were able to transform the recipient cells. Human primary fibroblasts engulfed the apoptotic bodies effectively within 30 minutes after co-cultivation. This mechanism is active and involves the actin cytoskeleton. *In situ* hybridization of transformed fibroblasts revealed the presence of HPV DNA in the nucleus of a subset of phagocytosing cells. These cells expressed the HPV16/18 E6 gene, which contributes to the disruption of the p53/p21 pathway, and the cells exhibited a tumorigenic phenotype, including an increased proliferation rate, polyploidy and anchorage independence growth. Such horizontal transfer of viral oncogenes to surrounding cells that lack receptors for HPV could facilitate the persistence of the virus, the main risk factor for cervical cancer development. This process might contribute to HPV-associated disease progression *in vivo*.

## Introduction

Epidemiological and experimental studies have highlighted that high-risk human papillomaviruses (HPV), especially HPV 16 and 18, play a major role in the induction of carcinomas of the cervix [1], [2]. The mechanistic aspects of HPV-induced carcinogenesis are most often related to deletion of the E2 ORF as a consequence of viral DNA integration into the host genome [3]. This leads to a deregulated expression of viral E6 and E7 genes, which represent the main transforming genes. At the heart of this transformation are the binding of E6 to p53 and E6AP, which favors p53 degradation [4] and the E7 complex formation with the retinoblastoma protein pRb [5], resulting in the deregulation of cell cycle control, DNA repair and apoptosis.

During tumor development, a large percentage of cells is lost through apoptosis [6]. Such cell death is triggered by a variety of extracellular signals, including growth/survival factor depletion, hypoxia and a loss of cell-matrix interactions, as well as intracellular signals such as DNA damage [7]. Finally, apoptotic cells are cleared by specialized phagocytic cells that inactivate and degrade their cellular components [8]. However, apoptotic cells can also be internalized by non-specialized recipient cells. Thus, fibroblasts are able to engulf apoptotic neutrophils [9], and liver endothelial cells can bind and phagocytose liver apoptotic bodies [10]. Through this endocytic process, apoptotic cells can act as a DNA vector, and the horizontally transferred DNA may confer a selective advantage to the recipient cell.

Horizontal gene transfer (HGT) has been well documented in prokaryotes and contributes to evolution, ecology and resistance to antibiotics [reviewed in [11], [12]]. While the horizontal transfer of genetic information between two eukaryotes has been reported in plants [13], [14] and invertebrates [15], few studies have focused on HGT between mammalian cells. The exchange of genetic information mediated by apoptotic bodies has been shown to occur between prostate cancer cells [16]. The apoptotic bodies of transformed lymphoid cells harboring integrated copies of the Epstein-Barr virus can also transfer viral DNA sequences [17]. Similarly, HIV-1 proviral genes are transferred to cells lacking receptors for viral entry [18]. DNA has also been reported to be transferred from apoptotic H-ras^V12^- and c-myc-transfected cells to p53−/− mouse embryonic fibroblasts, which leads to their transformation [19]. On the other hand, phagocytosing cells that express p53 or p21 are not transformed, suggesting a protective mechanism controlled by the p53 pathway [20]. Recently, Ehnfors J. *et al.* demonstrated that fibroblasts and endothelial cells are capable of acquiring and replicating H-ras^V12^ and c-myc DNA when apoptotic tumor cells contain the simian virus 40 large T (SV40LT) antigen [21]. These observations provided evidence that transformation efficiency is associated with the expression of SV40LT inhibiting p53 [22]. Because the majority of cervical carcinomas express the E6 viral oncoprotein, which promotes p53 degradation, as does SV40LT, we hypothesized that the horizontal transfer of HPV oncogenes could be an alternative mechanism of carcinogenesis.

Here, we present evidence that apoptotic cells derived from cervical-derived cancer cells harboring integrated copies of HPV are able to transform human primary fibroblasts (HPF). We further demonstrate that recipient tumor cells can be characterized by a high rate of proliferation and hyperploidy. In addition, the viral genetic material inhibiting the p53/p21 pathway is expressed in the transformed cells. To our knowledge, this is the first report of the transformation of human primary cells through the uptake of apoptotic bodies from HPV-infected cervical carcinoma cells.

## Results

### Apoptotic cervical carcinoma cells are internalized by fibroblasts

The apoptosis of cervical carcinoma donor cells was induced by UVB irradiation and staurosporine exposure as previously described [23], [24] and was documented by an analysis of phosphatidylserine exposure (annexin V staining), DNA content (propidium iodide staining) and nuclear fragmentation (DAPI staining) (Methods S1 and figure S1A and S1B). The treatment resulted in the absence of living cells capable of proliferation within the apoptotic cell suspensions (Methods S1 and figure S1C and SID). Previous studies have shown that apoptotic bodies derived from EBV-carrying B lymphocytes can transmit DNA by horizontal transfer and that EBV-integrated DNA may be preferentially transferred as compared with cellular DNA [17]. In this study, we questioned whether HPFs could engulf apoptotic cells derived from the cervical carcinoma cell lines HeLa (HPV18), Ca Ski (HPV16) and C-33 A (HPV-), regardless of virological status.

**Figure 1:**
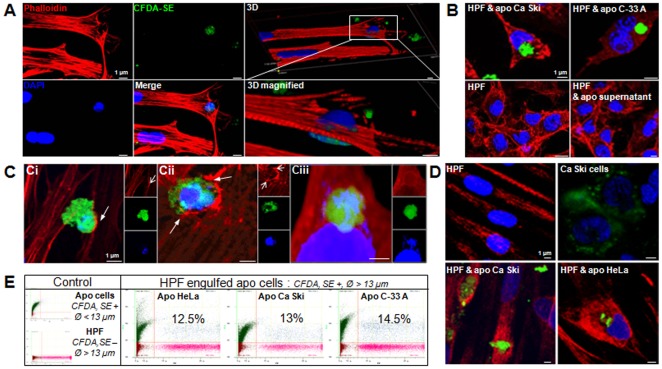
Apoptotic cells are engulfed by fibroblasts. (A) Co-culture of HPFs and apoptotic HeLa cells. (B) HPFs co-cultured with apoptotic Ca Ski cells (upper left), apoptotic C-33 A cells (upper right), alone (bottom left) and the supernatant of apoptotic HeLa cells (bottom right). (C) HPFs were cultured with apoptotic HeLa cells for different lengths of time: 1 h (Ci), 2 h (Cii) and 3 h (Ciii). The pictures show actin recruitment and membrane expansion formation (arrows). (D) HPFs, Ca Ski cells and HPFs plus apoptotic Ca Ski or HeLa cells were stained with anti-vimentin (TRITC) and anti-cytokeratin (Cy2) antibodies. Microscope observations were performed with a confocal microscope (scale bar: 1 µm). All results are representative of four independent experiments. (E) HPFs were incubated with apoptotic HeLa, Ca Ski or C-33 A cells for 48 h and apoptotic cell internalization was quantified by flow cytometry.

The presence of fluorescent apoptotic cells in the recipient cells was confirmed by confocal microscopy. Apoptotic HeLa cells containing DNA were entangled in the actin cytoskeleton of the HPFs within 48 h (figure 1A). Apoptotic Ca Ski and C-33 A cells were also taken up efficiently by the recipient (figure 1B). Incubation of the HPFs alone or with the supernatant of apoptotic cells did not result in CFDA, SE (5-(and 6-)-carboxyfluoresceine diacetate succinimidyl ester) staining, suggesting a link between green fluorescence and the presence of apoptotic cells (figure 1B). By tracking the fluorescent dyes at early time points (from 1 h to 3 h), we observed actin recruitment when apoptotic cells were bound to HPFs (figure 1Ci, white arrow). The fibroblast membrane expanded around both sides of the apoptotic cell through actin polymerization (figure 1Cii, white arrows). F-actin then surrounded the apoptotic cells to form a phagocytic cup and closed in a ring (figure 1Ciii). These microscopic observations are indicative of phagocytosis, although we have not specifically characterized this mechanism [25], [26]. Using specific markers of intermediate filaments for each cell type, we confirmed that the apoptotic cells were epithelial cells (cytokeratin positive) that were internalized by fibroblasts (vimentin positive) (figure 1D). Using the quantitative approach of flow cytometry, we assessed the percentage of HPFs that engulfed the stained apoptotic carcinoma cells. Regardless of the type of apoptotic cells used, the internalization efficiency was similar (12.5% with apoptotic HeLa; 13% with apoptotic Ca Ski; 14.5% with apoptotic C-33 A) (figure 1E). However, we noted that 12 to 15% of the fibroblasts were able to take up the apoptotic cells, while the number of apoptotic cells seeded was ten times larger than that of the HPFs. This suggests that fibroblasts have a limited potential in the efficiency and/or quantity of apoptotic cell internalization. When recipient cells were co-incubated with apoptotic cells at 4°C for 48 h, the percentage of internalization dropped significantly to 2%, thus suggesting that the internalization process follows an energy-dependent pathway (figure S2). These results indicate that human primary fibroblasts can engulf apoptotic cells, independent of their virological status, through phagocytosis.

### Only HPV-positive apoptotic cells efficiently transform recipient cells

**Figure 2:**
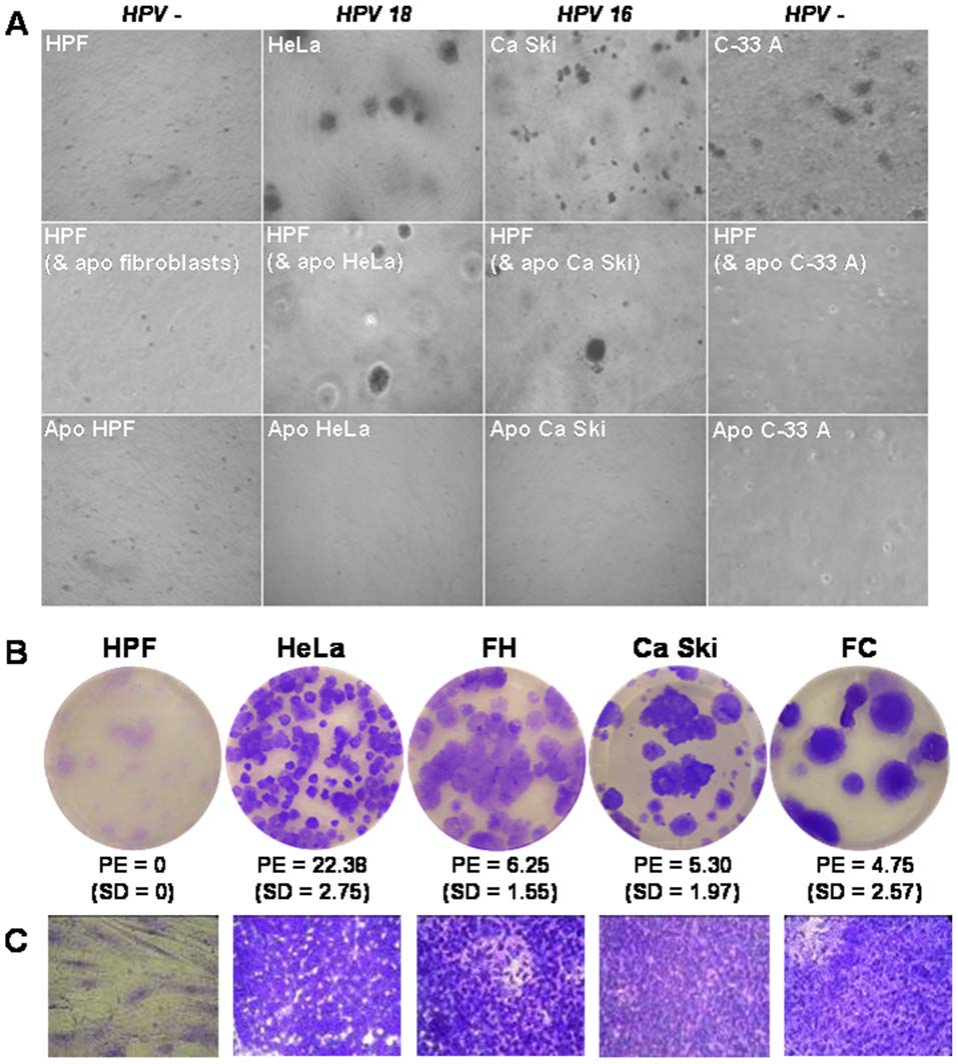
Apoptotic cells infected by HPV transform fibroblasts. (A) HPFs and HeLa, Ca Ski and C-33 A cervical cancer cells (upper line); HPFs after 48 h of exposure to apoptotic cells (apo HPF, apo HeLa, apo Ca Ski, apo C-33 A) (middle line) were cultured in soft agar for 21 days. Apoptotic cells alone were also cultured as a control (lower line). Photographs were taken with a 20× magnification lens. (B and C) HPFs, HeLa cells, Ca Ski cells and transformed fibroblasts by apoptotic HeLa (FH) and apoptotic Ca Ski (FC) cells (after selection on soft-agar), were grown at a limit-dilution for 21 days. (B) The colonies stained with purple crystal were counted, and the plating efficiency (PE, percentage of cells able to form colonies; SD, standard deviation) was calculated. (C) Colony magnifications were photographed with a 40× magnification lens.

Ehnfors *et al.* demonstrated that DNA from rat fibrosarcoma apoptotic cells transfected with H-*ras* ^V12^, c-*myc* and SV40LT is transferred to and transforms primary fibroblasts [21]. Because HPV oncogenes, like SV40LT, are capable of efficiently transforming infected cells and blocking the p53 pathway, among other effects, we tested whether fibroblasts cultured with apoptotic cells were able to grow with anchorage independence by measuring their ability to form colonies in a soft agar assay, as observed with the HeLa, Ca Ski and C-33 A cancer cells (figure 2A, upper line). In contrast, human primary fibroblasts were unable to grow without a solid matrix (figure 2A, upper line). Although the three types of apoptotic bodies were internalized by fibroblasts, only the apoptotic cells harboring HPV (HeLa and Ca Ski cells) transformed the HPFs (figure 2A, middle line). The controls with apoptotic cells alone did not result in colony formation (figure 2A, lower line). These findings demonstrate that the incubation of apoptotic cells with HPV-integrated DNA effectively induced anchorage-independent growth of HPFs, a hallmark of transformation and an *in vitro* correlate of tumorigenicity *in vivo* [27]. The transformation status of the HPFs was further tested by limit-dilution assays. Indeed, in contrast to primary fibroblasts, the fibroblasts transformed by apoptotic HeLa (FH) and Ca Ski (FC) cells had the ability to form multilayer colonies when they were grown at low density (figure 2B). At this stage, the FH and FC began to exhibit a transformed phenotype, with some of the cells appearing rounded, unlike the control HPFs, which displayed a spindle shape (figure 2C). Moreover, the transformed fibroblasts consisted mostly of packed or aggregated small cells such as HeLa and Ca Ski cells, whereas the primary fibroblasts formed a flattened monolayer.

**Figure 3:**
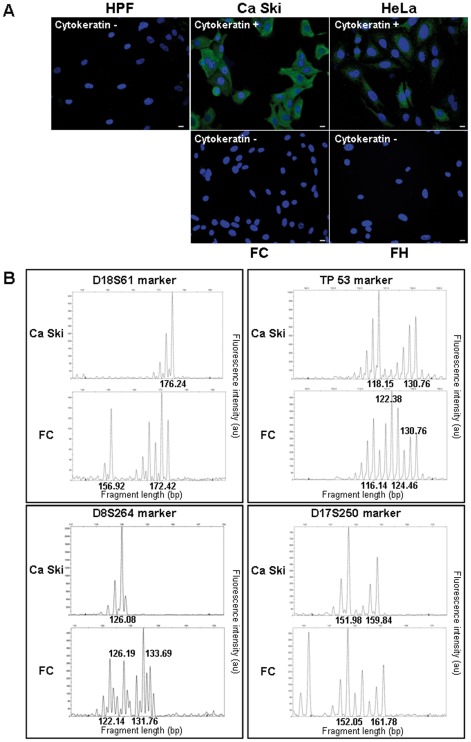
Cytokeratin expression and DNA profiles of transformed fibroblasts differ from the parental cancer cells. (A) HPFs, Ca Ski, HeLa, FC and FH cells were analyzed by immunocytofluorescence for cytokeratine expression (scale bar: 1 µm). (B) DNA from Ca Ski and FC cells was amplified by fluorescent PCR using 20 microsatellites. Four representative microsatellite amplifications are shown. Different types of allele are observed using four markers: D18S61 TP53, D8S264 and D17S250.

Figure 3A shows that transformed fibroblasts, FC and FH, are not positive for cytokeratin staining, unlike epithelial HeLa and Ca Ski cells, revoking the possibility that the observed transformed cells are rare surviving cancer cells and a clonal evolution of Ca Ski cells. As illustrated in the upper left panel of figure 3B (D18S61 marker, an example of 20 markers of DNA typing used), transformed fibroblasts have a pattern of DNA that is different from the original tumor cells. Other results of DNA typing experiments showed that FC cells were different from Ca Ski cells but contain some alleles from Ca Ski DNA (TP53 and D8S264 markers), reflecting the transfer of small DNA fragments. FC DNA also contains parts of chromosomes (D17S250 markers), reflecting the transfer of large DNA fragments as described by Holmgren [17], [19]. Although these results demonstrate the chromosomal rearrangement (loss and gain of alleles) of FC cells, we cannot exclude the possibility of cross-contamination, despite this being highly unlikely.

**Figure 4:**
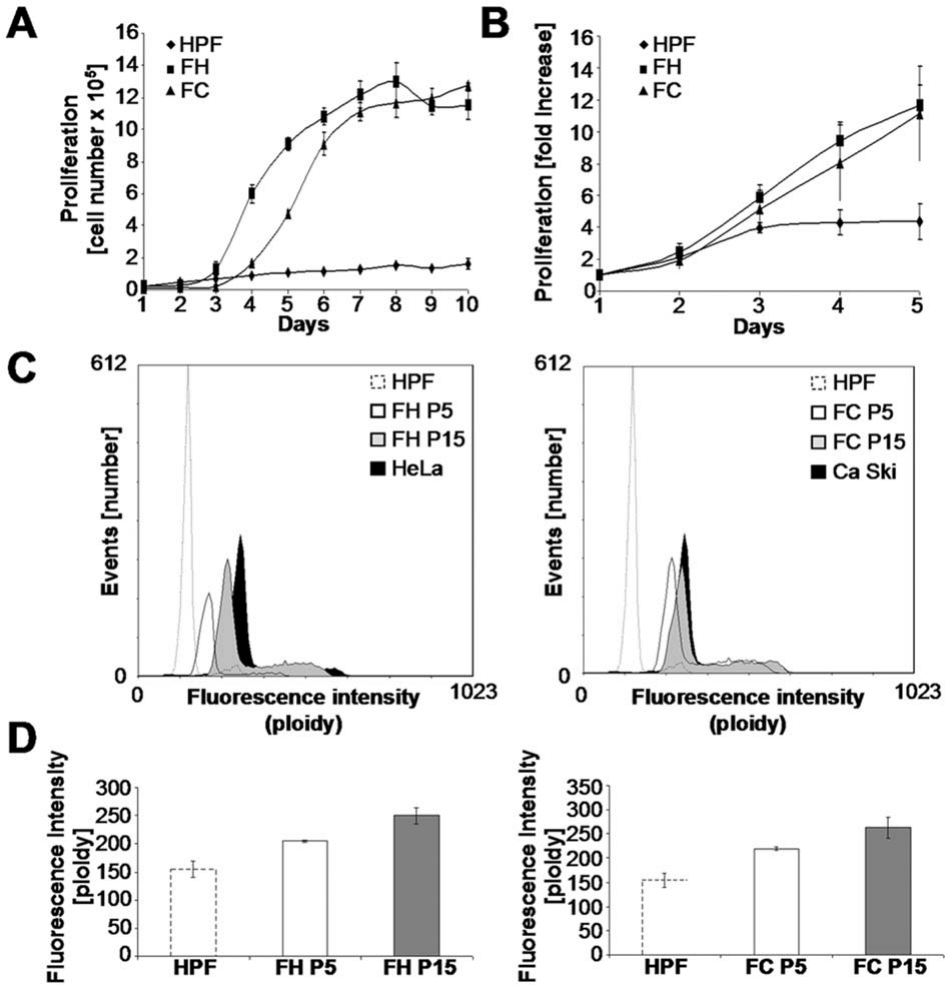
Transformed fibroblasts acquire tumorigenic characteristics. (A and B) HPFs and HPFs transformed by apoptotic HeLa (FH) or apoptotic Ca Ski (FC) cells were grown for the indicated lengths of time. The cell proliferation was monitored each day by counting the total numbers of cells (A) and by MTT assays (B). The graphs present the mean (+/− SD) of three independent experiments. (C) HPFs, FH and FC at passages 5 (P5) and 15 (P15) were stained (10^6^ cells) with propidium iodide solution, and analyzed by flow cytometry. (D) The MFI of the G0/G1 peak (mean of three independent experiments +/− SD) displays the ploidy of HPF, FH and FC at P5 and FH and FC at P15.

Next, we determined the effects of HPF transformation on the proliferation rate using a growth curve and the results from the MTT (3-[4,5-dimethylthiazol-2-yl]-2,5-diphenyl tetrazolium bromid) proliferation assay shown in Fig. 4A and 4B, respectively. The FH had an average population doubling time of 15 h, and the FC had an average time of 16 h, while the HPF doubling time was greater by a factor of 19 (298 h) (figure 4A). The mitochondrial activity measured by the MTT proliferation assay was concordant with the growth curve results (figure 4B). These growth rate modifications support the tumorigenic potential of the newly transformed fibroblasts. We therefore tested whether the increased growth rate and the transformation of the recipient cells were associated with genetic modifications leading to hyperploidy. Cytometry assays showed that the DNA content increased with the passages of transformed fibroblasts (figure 4C). In our experiments, an HPF mean fluorescence intensity (MFI) of 154 corresponded to diploid cells (figure 4D). The MFI increased to 205 and 250 after 5 (P5) and 15 (P15) passages of FH cells, respectively. We observed similar results for the FC cells (219 at P5 and 264 at P15). The MFI at passages 15 of FH and FC represented hypertriploidy. Aneuploidy, as seen in our model, is often caused by a particular type of genetic instability and is one of the most common properties of cancers [28].

### Apoptotic HPV-associated cancer cells efficiently transfer viral oncogenes to fibroblasts

**Figure 5:**
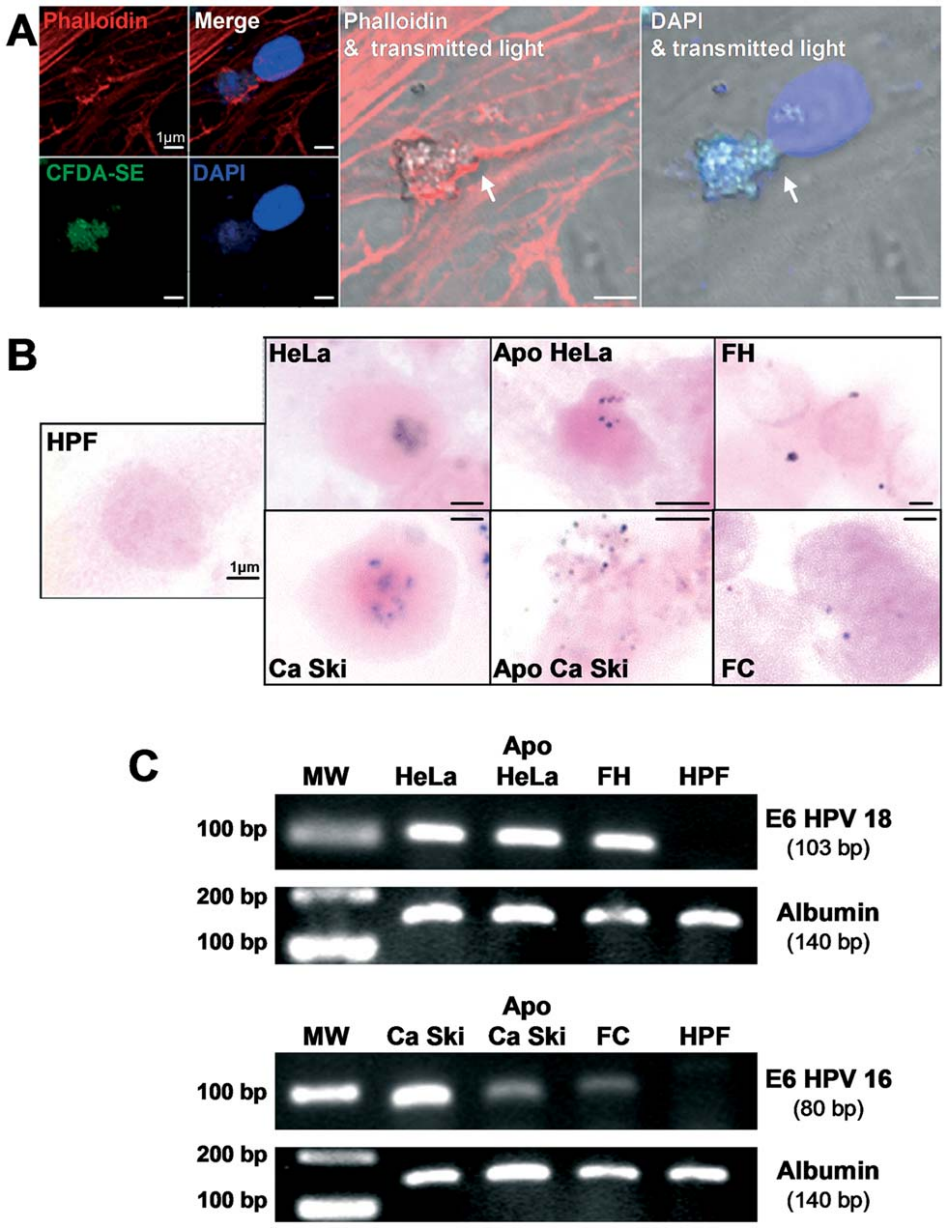
HPV oncogenes are transferred through apoptotic cells to transformed fibroblasts. (A) The images illustrate the DNA transfer from the apoptotic cells to the fibroblast recipients (arrows). Microscopic observations were performed with a confocal microscope (scale bar: 1 µm). (B) High-risk HPV DNA was detected by *in situ* hybridization in parental and apoptotic HeLa and Ca Ski cells, FH and FC but not in HPFs. Cells were counterstained with eosin (scale bar: 1 µm). (C) Agarose gel electrophoresis of amplified human albumin and E6 HPV18 and E6 HPV16 DNA (MW: molecular weight). The images are representative of three independent experiments.

**Figure 6:**
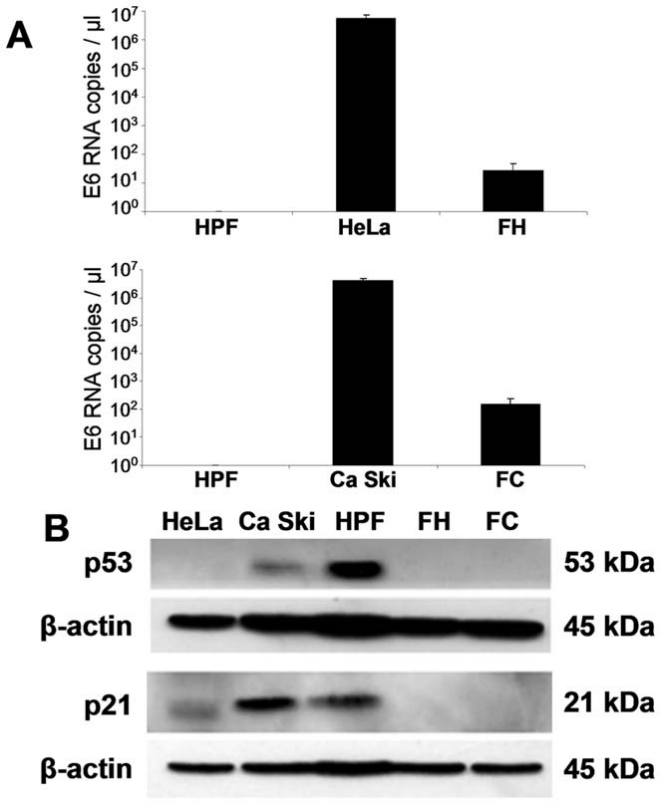
Downregulation of p53 in transformed fibroblasts expressing E6 HPV transcripts. (A) E6 HPV18 and E6 HPV16 RNA quantification from HPFs, parental cells and fibroblasts transformed by apoptotic HeLa cells or apoptotic Ca Ski cells (named FH and FC respectively) was performed by real-time quantitative PCR following reverse transcription. The graphs represent the mean of three independent experiments (+/− SD). (B) Immunoblotting analyses of p53 and p21 expression in cancer cell lines, HPFs and FH and FC transformed fibroblasts, using mouse monoclonal antibodies against p53 and p21. Blots were also probed with a β-actin antibody.

Because only apoptotic HeLa and Ca Ski donor cells were able to transform fibroblasts, we hypothesized that HPV oncogenes could be transferred to the recipient cells. Our confocal microscopy analysis suggested that genetic material was transferred from the apoptotic cells to the fibroblast nuclei after 6 h of co-culture (figure 5A, white arrows). The actin cytoskeleton reorganization appeared to deliver the apoptotic cell toward the nucleus for DNA transfer, as seen at a high magnification by superimposing transmitted light with phalloidin or DAPI staining images. Following these observations, we investigated the HPV DNA transfer in fibroblast recipients using *in situ* hybridization (ISH) with a probe hybridized specifically to high-risk HPV DNA. HeLa and Ca Ski cells were used as positive controls. Confirming our hypothesis, the hybridization signals were observed as purple dots in the apoptotic cells and nuclei of the transformed fibroblasts (FH and FC) whereas no signal was detected in the HPFs (figure 5B). These data validate the hypothesis of horizontal transfer of viral oncogenes. A second approach consisting of amplifying the E6 DNA of HPV 16 and 18 confirmed the presence of viral DNA in the transformed fibroblasts (figure 5C). We further analyzed the expression of E6 HPV16 and E6 HPV18 by reverse transcriptase followed by real-time quantitative PCR. Figure 6A illustrates that the E6 transcripts were detected in the transformed FH and FC cells with however lower levels than in the parental HeLa and CaSki cells. These data suggest that the transfer of viral oncogenes is efficient and functional. To more thoroughly scrutinize the role of the transferred E6 oncogenes as inhibitors of p53 expression, we immunoblotted for p53 and one of its targets, p21. Accordingly, the p53 and p21 levels of the transformed HPFs decreased substantially, similar to the decrease in donor cancer cells (figure 6B). Overall, these results emphasize a critical role of viral oncogene transfer in the transformation of primary cells, a process that bypasses the p53 pathway.

## Discussion

The results of this study provide direct evidence of the oncogenic potential of HPV positive apoptotic cells. The transfer of viral DNA derived from HPV-positive cervical cancer cells through HGT promoted the growth and transformation of HPFs and could represent an alternative mechanism for HPV-associated cellular transformation. Bergsmedh *et al.* previously demonstrated horizontal oncogene transfer between eukaryotic cells [19]. In their model, the donor cells were primary rodent fibroblasts modified to overexpress c-*myc* and H-*ras* ^V12^ and a hygromycin-resistant gene that permitted a highly stringent selection of transformed recipient cells after HGT.

In our study, the internalization rates of apoptotic cancer cells were similar, regardless of the HPV status. However, the finding that only HeLa and Ca Ski cancer cells carrying naturally integrated HPV oncogenes, but not HPV-negative C-33 A cells are able to transform phagocytosing fibroblasts provides support for the hypothesis that viral DNA transferred by apoptotic cells can be reused and expressed by recipient cells.

The *in vitro* recipient cell transformation by horizontally transferred DNA, facilitated by DNA fragmentation, was shown to be dependent on p53 [29]. Indeed, p53- or p21-deficient cells, but not wild-type p53 fibroblasts, became tumor-like after their uptake of c-*myc* and H-*ras* ^V12^ oncogenes, indicating that the Chk2/p53/p21 signaling pathway protects cells against the propagation of potentially harmful DNA [19], [20], [30]. Moreover, SV40LT, which facilitates p53 degradation, can overcome this genetic surveillance both *in vitro* and *in vivo* [21]. Like SV40LT, the E6 oncoproteins of HPV type 16 and 18 are physically associated with wild-type p53 and favor its proteosomal degradation [reviewed in [31]]. An analysis of the transformed fibroblasts revealed that they contained E6 HPV16 or E6 HPV18 DNA. In addition, ISH showed that HPV DNA from the apoptotic cancer cells was transferred into the nuclei of recipient fibroblasts. Once the DNA was transferred, the expression of HPV E6 genes was detected at the RNA level up to 15 weeks after the start of co-culture experiments. The E6-expressing recipient cells exhibited decreased levels of p53 as well as its target, p21, which might partly explain the alterations in growth control circuitry.

We intended to confirm by genotyping that transformed fibroblasts were unique and were derived from primary fibroblasts and parental cancer cell lines. By studying several microsatellites, we found that they actually harbored some alleles identical to those identified in apoptotic cervical cancer cells. However, due to the loss of primary fibroblasts to senescence, we were unable to confirm that they were derived from primary fibroblasts. We cannot therefore completely exclude the possibility of cross-contamination with an unrelated cell line, even if this possibility appears improbable. However, virally altered fibroblasts that were able to form colonies demonstrated peculiar morphological characteristics, a high proliferation rate and aneuploidy. The transition from diploidy to aneuploidy, a hallmark observed in virtually all cancers [28], has been noted in early high-risk HPV-associated lesions of the cervix [32] and has been attributed to the synergistic effect of E6 and E7 oncoproteins [reviewed in [31]]. However, high-risk HPV immortalized cells are non-tumorigenic, and the activation of cellular oncogenes c-*myc*, H-*ras* and c-*fos* is necessary to completely overcome the anti-oncogenic function of p53 and to result in cervical cancer development [33], [34], [35]. Further study of the expression of these possibly activated cellular oncogenes will aid in understanding the mechanism of fibroblast transformation. Nonetheless, HPV oncogene transmission could have a role more crucial than considered, since the expression of HPV16 E6 oncogene in HPV negative C-33 A cells confers an aggressive phenotype as shown by the radiation resistance in transplanted tumors [36].

Escape from immune surveillance mechanisms may represent the main risk factor for HPV DNA persistence and lesion progression, whereas the viral transfer from apoptotic bodies to surrounding cells lacking receptors for HPV *in vivo* could facilitate the persistence of HPV in the cervix. Moreover, such transfer might explain the spread of HPV to mesenchymal cells, as observed by ISH in cervical carcinomas [37], [38], and the possibility of a stromal reservoir for HPV. Furthermore, in human solid tumors, a subset of cancer cells, called cancer stem cells, that are likely initiated as a result of HGT cause very aggressive cancers with a high propensity toward metastatic dissemination [39]. This might explain the positive association between the rate of intratumoral apoptosis and several cervical tumor parameters such as tumor size, lesion grade, metastatic phenotype and patient survival [7], [40], [41], [42].

In addition, the increased prevalence of apoptotic cells following chemo- and/or radiation therapy could be partly responsible for the recurrence of HPV lesions by inducing the transformation of new cells at the same or different anatomic locations months or years after remission [43], [44]. Investigations of this possibility are warranted, in addition to efforts to find new therapeutic strategies targeting E6/E7 oncogenes to limit their horizontal transfer and to control tumor development.

## Materials and Methods

### Cell culture and apoptosis induction

HPFs were isolated from adult human skin after abdominoplasty as previously described [45] and were grown in complete DMEM (Lonza) containing 10% FBS (Lonza), penicillin/streptomycin and L-glutamine. HPFs extracted from surgical residues are not subject to validation from an ethics committee and the patient's consent in accordance with the law L.1245-2 of the “Code de la santé publique” applied in France. Moreover, the laboratory of skin engineering of Prof. Philippe Humbert's dermatology departement, providing human fibroblasts, has manuscript documents stating the patient's non-opposition to the use of his surgical residues to medical research in accordance with the law L.1211-2. Human cervical carcinoma cell lines (ATCC), HeLa (HPV18, wild-type p53) and C-33 A (HPV negative, mutated p53) were grown in complete EMEM (Lonza), and Ca Ski cells (HPV16, wild-type p53) were grown in complete RPMI (Lonza). They were monitored monthly and found to be free of mycoplasms. Twelve hours prior to apoptosis induction, the carcinoma cells were seeded at 2×10^4^ cells/cm^2^. They were then treated with 20 mJ/cm^2^ UVB irradiation followed by 300 nM staurosporine (STS) (Sigma Aldrich) for 48 h. The apoptotic cells were harvested after centrifugation of the supernatant at 300 g for 10 min. The apoptosis detection was conducted as described in the supplemental information (Methods S1). The apoptotic cells were incubated with HPFs at a ratio of 10∶1. This ratio was chosen because a higher ratio causes fibroblast death and a lower ratio decreases the rate of internalization.

### Apoptotic cell internalization analysis

The apoptotic cells were stained with 1 µg/ml CFDA, SE (Invitrogen Ltd) diluted in DMEM with 2% FBS for 13 min at 37°C. After washing, they were incubated for 48 h with recipient cells. For cytometry analysis, the fibroblasts incubated with the apoptotic cells were harvested by trypsinization and analyzed using a Cell Lab Quanta™ SC flow cytometer (Beckman Coulter). Apoptotic cells were labeled with CFDA, SE and HPFs were distinguished from apoptotic cells by their diameter as evaluated by flow cytometry. Events with small diameters (<13 µm) and positive for CFDA SE, were considered apoptotic cells;, events with large diameters (>13 µm) and negative for CFDA, SE, were HPFs, and events with large diameters and positive for CFDA, SE, were HPFs with engulfed apoptotic cells.

For confocal microscopy, fibroblasts grown on coverslides were fixed with 3.7% paraformaldehyde for 20 min at 4°C and permeabilized using 0.1% triton X100 for 10 min at room temperature (RT). The cells were stained using TRITC (tetramethylrhodamine-isothiocyanate)-conjugated phalloidin (Sigma Aldrich) for 30 min at 4°C and with 300 nM 4′,6-diamidino-2-phenylindole, dihydrochloride (DAPI; Invitrogen) for 5 min at RT. The 3D picture (z projection) was reconstituted from 32 horizontal 2D slices obtained by confocal microscopy. Another set of cells was incubated with two primary antibodies: a monoclonal mouse anti-human cytokeratin antibody (clone AE1/AE3, Dakocytomation) used at 1∶50 and a monoclonal rabbit anti-human vimentin antibody (clone SP20; GeneTex) used at 1∶100 dilution. Corresponding secondary antibodies (Jackson ImmunoResearch) coupled to cyanine 2 (CY™2-conjugated AffiniPure Goat anti-Mouse IgG) or to rhodamine (Rhodamine (TRITC)-conjugated AffiniPure Donkey Anti-Rabbit IgG) were used at 1∶50 dilution. The coverslides were finally examined by using an Olympus FluoView 1000 fluorescence microscope (Olympus).

### Colony formation assay, cell growth and aneuploidy analysis

Soft agar assays were conducted in 24-well plates in semi-solid media (DMEM, 10% FBS, 0.35% agar; Invitrogen) with 8×10^3^ and 3×10^5^ cells/ml on a media base layer (RPMI, 10% FBS, 0.5% agar). The cells were grown for 21 days, and the colonies were observed using an Axiovert 25 inverted microscope (Zeiss) [27]. The colonies were then harvested by scraping the surface of the soft-agar and two cell lines of fibroblasts transformed by apoptotic HeLa (FH) and Ca Ski (FC) cells were derived and used for further experiments.

Primary fibroblast transformation was also tested by limit-dilution cultures in 6-well plates at 5×10^2^ cells/well for 21 days. The colonies were then stained using a purple crystal solution (0.1% purple crystal (w/v), ethanol 5%) and photographed with a Nikon Coolpix 4500 digital camera (Nikon) [46].

The proliferation of the transformed cells was monitored by counting the total number of cells in each individual well daily for 10 days with a Cell Lab Quanta™ SC flow cytometer. The proliferation was also monitored using the MTT test with the Cell Proliferation Kit I (MTT) (Roche) for 5 consecutive days. The formation of purple formazan crystals was quantified using the scanning multi-well spectrofluorimeter EnVision® 2102 Multilabel Reader (Perkin Elmer). For the aneuploidy analysis, 10^6^ living cells were collected and fixed overnight in 70% (v/v) cold ethanol. After two washes, the cells were stained with a propidium iodide solution (0.1 mg/ml propidium iodide, Sigma Aldrich; 20 µg/ml RNaseA DNase-free, ABgene). After 15 min at RT, 20,000 events were analyzed by an FC500 flow cytometer (Beckman Coulter). The mean fluorescence intensity (MFI) of the G0/G1 peak was evaluated using the CXP™ cytometer software and expressed in arbitrary units.

### *In situ* hybridization

Cells were cultured overnight on poly-L-lysine microscope slides (Thermo Fisher Scientific), fixed with 3.7% paraformaldehyde for 15 min at RT and permeabilized with PBS Tween 1% for 10 min at RT. The high-risk HPV DNA detection was conducted using the INFORM® HPV family probe (Ventana Medical Systems) and the BenchMark® XT Automated Slide Stainer (Ventana Medical Systems) as described by the manufacturer. The cells were also counterstained with eosin.

### Amplification of microsatellites and viral genes

Total DNA was extracted from 10^6^ cells using the QIAamp® DNA Mini Kit (Qiagen, Courtaboeuf, France). For typing, extracted DNA was amplified by fluorescent PCR as described previously [47]. A panel of 20 microsatellites was used. Primers were obtained from the Genome Data Base (www.gdb.org) or Genemap'99 (www.ncbi.nlm.nih.gov/genemap99/). Amplified fragments were analyzed on an ALF Sequencer (Amersham-Pharmacia®, Piscataway, NJ, US), allowing for a very sensitive and quantitative evaluation of the allele ratio by measuring the peak height of both alleles.

For viral gene detection, PCRs targeted the albumin, E6 HPV16 and E6 HPV18 genes, using 500 nM of the corresponding primers (Eurogentec, Seraing, Belgium). The sequences of the albumin and E6 HPV16 primers have been previously described by Laurendeau *et al.* and Saunier *et al.*, respectively, while the sequences of the E6 HPV18 primers are patent pending [48], [49]. After a hot-started reaction at 94°C for 5 min, the target DNAs were amplified for 30 cycles for 30 sec at 94°C, 30 sec at the annealing temperature (57°C for albumin, 51°C for E6 HPV16 and 55°C for E6 HPV18) and 20 sec at 72°C, followed by a 7-min extension at 72°C. The PCR products were analyzed by agarose gel electrophoresis.

### Detection of viral transcripts

Total RNA was extracted from 2×10^6^ cells using the QIAamp® RNA Blood Mini Kit (Qiagen). After DNAse I treatment (Invitrogen), the reverse transcription of 500 ng of RNA was performed using the MMLV-Reverse Transcriptase (Invitrogen). Twenty five nanograms of cDNA were pre-amplified using 45 nM E6 HPV16, E6 HPV18 and human β-2-microglobulin primers in the TaqMan PreAmp Master Mix (Applied Biosystems) as follows: a 10-min step at 95°C and 10 amplification cycles (15 sec at 95°C, 4 min at 60°C). The quantification of the pre-amplified products was performed with a 7500 Real Time PCR System (Applied Biosystems) in the TaqMan Gene Expression Master Mix (Applied Biosystems), using 500 nM of each primer (Eurogentec) and TaqMan Probes (100 nM for β-2-microglobulin: 5′FAM-cctccatgatgctgcttacatgtctcgatccc-BHQ1-3′; 250 nM for E6 HPV16: 5′-FAM-aggagcgacccagaaagttaccacagtt-BHQ1-3′ or E6 HPV18: 5′-JOE-caacacggcgaccctacaagctacc-BHQ1-3′, Eurogentec) according to the manufacturer's thermal cycling protocol. Standard curves were obtained by serial dilutions over a range of six log concentrations of the pBR322-HPV16 and pBR322-HPV18 diluted in 50 ng/µl salmon sperm DNA.

### Immunoblotting

Immunoblotting was performed as described previously [50]. Briefly, 30 µg of protein was separated by SDS-PAGE and electrotransferred onto Hybond™-P membranes (GE Healthcare Lifesciences). Mouse monoclonal antibodies against p53 (clone DO-7, Becton Dickinson), p21 (clone 6B6, Becton Dickinson) and β-actin (clone AC-15, Sigma Aldrich) were used at 1∶500, 1∶500 and 1∶8000 dilutions, respectively. A secondary antibody coupled with alkaline phosphatase was used at 1∶10000 for p53, 1∶5000 for p21 and 1∶40000 for β-actin. The immunoblots were analyzed by chemiluminescence using the ECL Plus Reagents System (GE Healthcare Wauwatosa).

## Supporting Information

Figure S1 **UVB and staurosporine treatments of HeLa, Ca Ski and C-33 A cells induce the formation of apoptotic body suspensions devoid of living cells.** HeLa, Ca Ski and C-33 A cells were treated with UVB irradiation at 20 mJ/cm^2^ (UV 20) and/or 300 nM staurosporine (STS) for 48 h. The cells and apoptotic body suspension were harvested and characterized. Ai, Phosphatidylserine exposure was evaluated by flow cytometry after annexin V-FITC labeling. Aii, The DNA content was quantified using propidium iodide staining. (A) Cumulative data of four independent experiments (upper panel) and representative data of flow cytometry analysis (lower panel). (B) Nuclear fragmentation was observed by fluorescent microscopy after DAPI staining. The proliferation of the cells and apoptotic body suspensions was verified by MTT (C) and by culture from 7 to 28 days (D). For each panel, four independent experiments were performed yielding similar results.
(TIF)

Figure S2 **Apoptotic cell engulfment involves an energy-dependent pathway.** HPFs were incubated with apoptotic HeLa (Apo HeLa), Ca Ski (Apo Ca Ski) or C-33 A (Apo C-33 A) cells for 48 h at 37°C and 4°C. Apoptotic cells were labeled with CFDA, SE, prior to incubation. HPFs were distinguished from apoptotic cells by their diameter as evaluated by cytometry (> or <13 µm). Events with small diameters and positive for CFDA, SE, were considered apoptotic cells (upper left quadrant), events with large diameters and negative for CFDA, SE, were HPFs (bottom right quadrant), and events with large diameters and positive for CFDA, SE, were HPFs with engulfed apoptotic cells (upper right quadrant). The results are representative of three independent experiments.
(TIF)

Methods S1 (DOC)
