# Supplementary figures and images for: Apoptotic HPV Positive Cancer Cells Exhibit Transforming Properties
Source: PLoS One. 2012 May 4;7(5):e36766. doi: 10.1371/journal.pone.0036766 (PMC3344932; doi:10.1371/journal.pone.0036766)

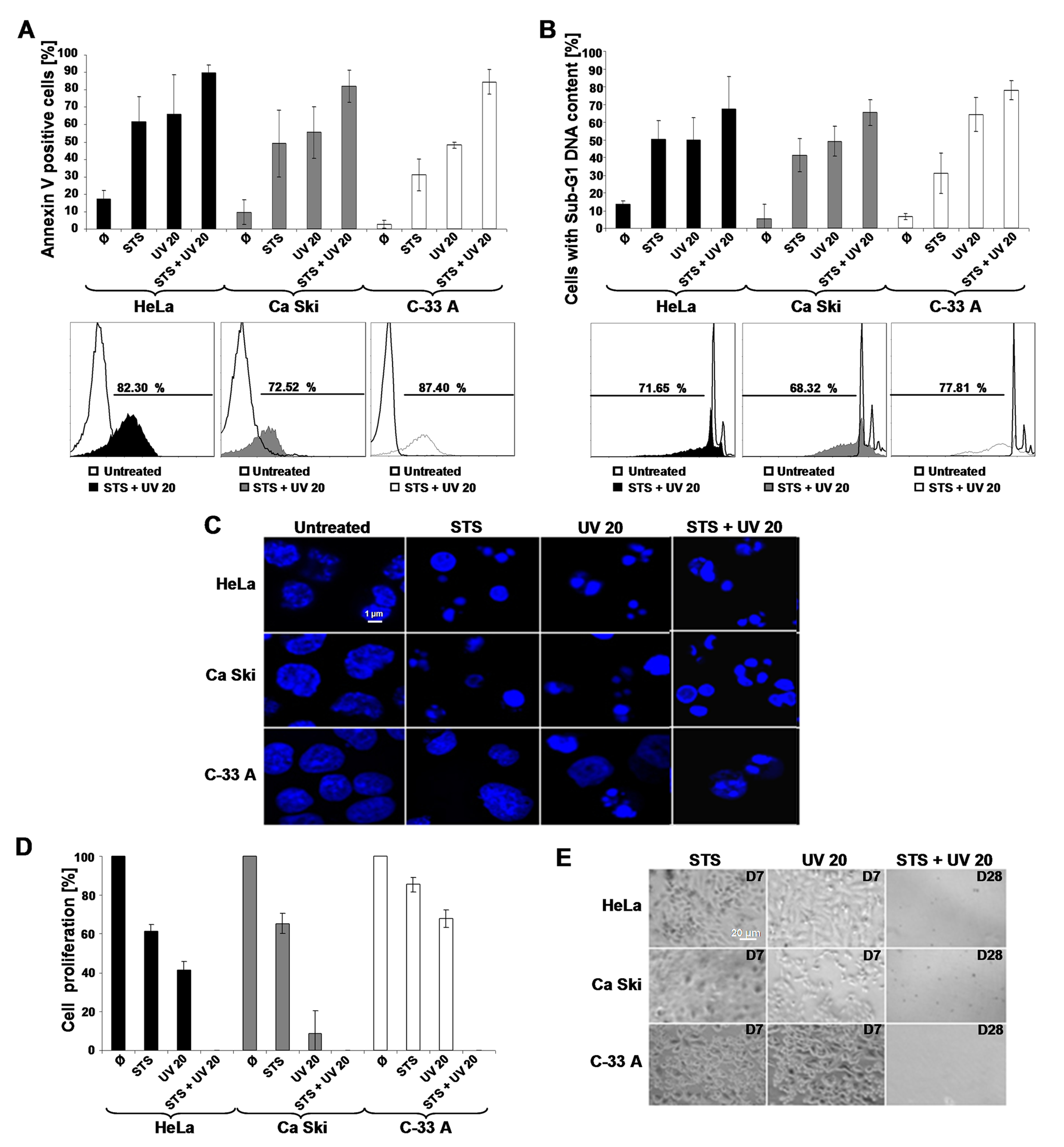

Supplement: Figure S1 — UVB and staurosporine treatments of HeLa, Ca Ski and C-33 A cells induce the formation of apoptotic body suspensions devoid of living cells. HeLa, Ca Ski and C-33 A cells were treated with UVB irradiation at 20 mJ/cm2 (UV 20) and/or 300 nM staurosporine (STS) for 48 h. The cells and apoptotic body suspension were harvested and characterized. Ai, Phosphatidylserine exposure was evaluated by flow cytometry after annexin V-FITC labeling. Aii, The DNA content was quantified using propidium iodide staining. (A) Cumulative data of four independent experiments (upper panel) and representative data of flow cytometry analysis (lower panel). (B) Nuclear fragmentation was observed by fluorescent microscopy after DAPI staining. The proliferation of the cells and apoptotic body suspensions was verified by MTT (C) and by culture from 7 to 28 days (D). For each panel, four independent experiments were performed yielding similar results. (TIF) [file pone.0036766.s001.tif]

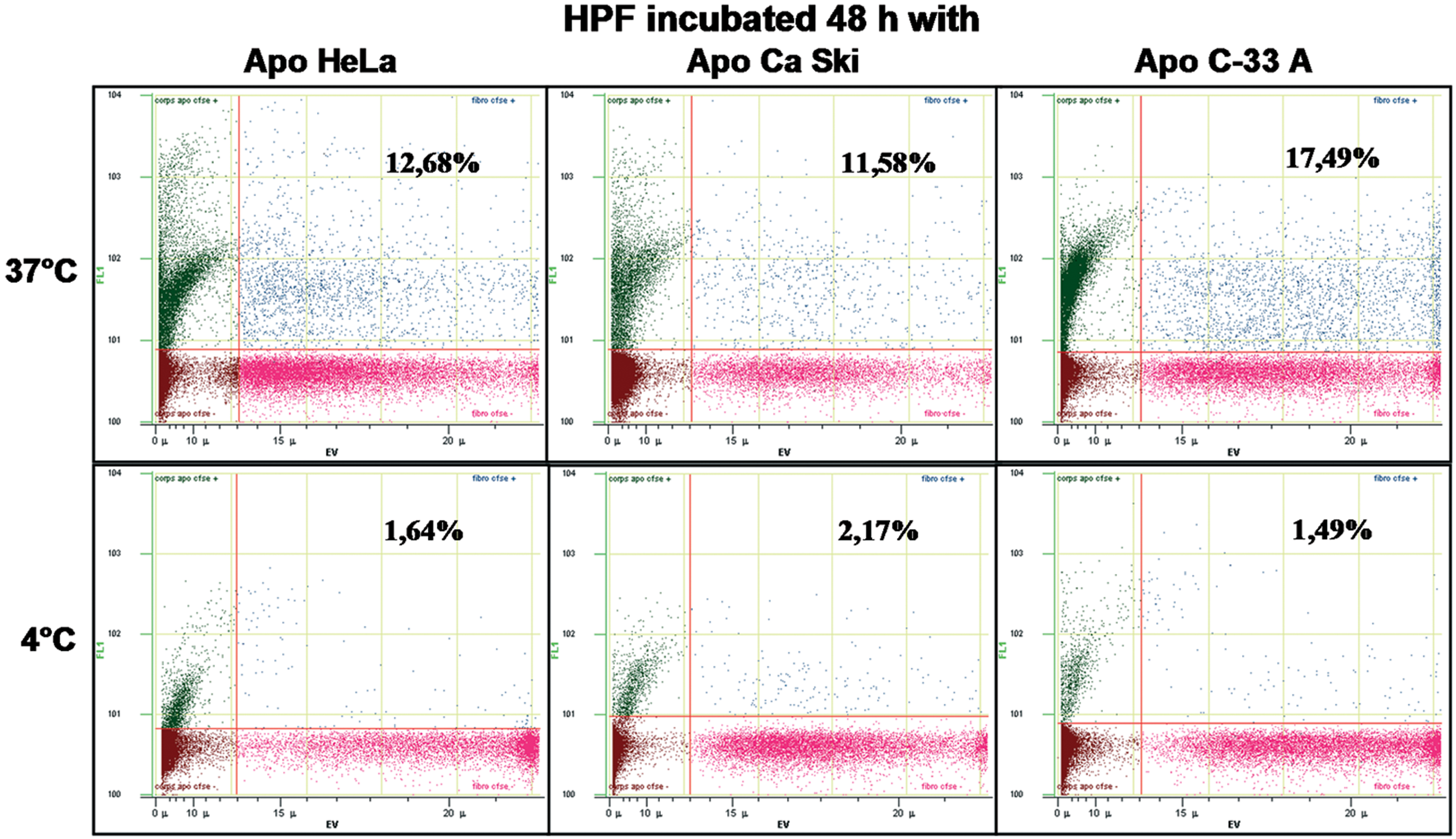

Supplement: Figure S2 — Apoptotic cell engulfment involves an energy-dependent pathway. HPFs were incubated with apoptotic HeLa (Apo HeLa), Ca Ski (Apo Ca Ski) or C-33 A (Apo C-33 A) cells for 48 h at 37°C and 4°C. Apoptotic cells were labeled with CFDA, SE, prior to incubation. HPFs were distinguished from apoptotic cells by their diameter as evaluated by cytometry (> or <13 µm). Events with small diameters and positive for CFDA, SE, were considered apoptotic cells (upper left quadrant), events with large diameters and negative for CFDA, SE, were HPFs (bottom right quadrant), and events with large diameters and positive for CFDA, SE, were HPFs with engulfed apoptotic cells (upper right quadrant). The results are representative of three independent experiments. (TIF) [file pone.0036766.s002.tif]
