# Supplementary material for: Apoptotic HPV Positive Cancer Cells Exhibit Transforming Properties
Source: PLoS One. 2012 May 4;7(5):e36766. doi: 10.1371/journal.pone.0036766 (PMC3344932; doi:10.1371/journal.pone.0036766)
Supplement: Methods S1 — (DOC) [file pone.0036766.s003.doc]

**Methods S1**

**Apoptosis analysis.** For phosphatidylserin exposure analysis, apoptotic cell suspension was analysed with annexin V-FITC apoptosis detection kit I (Becton Dickinson) according to the manufacturer’s instructions. Twenty thousand events were collected for each sample and analysed by cytomics FC500 flow cytometer (Beckman Coulter) with CXP^TM^ cytometer software.

For nuclear fragmentation observation by microscopy, apoptotic cells in the media were retained and pooled with remaining adherent cells that were harvested by trypsinization. They were washed twice with PBS, centrifuged at 300g for 10 min, resuspended and fixed by 3.7% formaldehyde for 20 minutes at 4°C. Fixed cells were stained with 300 nM DAPI for 5 min at RT, washed twice with PBS, centrifuged at 300g for 10 min and resuspended in fluorescent mounting media (Dakocytomation). One drop of suspension was applied to a poly-L-lysin microscope slide (Thermo Fisher scientific), cover with a coverslip and observed with Olympus FluoView 1000 fluorescent microscope (Olympus).

**Proliferation test.** In order to determine whether living cells were present in suspension of apoptotic cells, those suspensions were cultured in complete medium during 28 days and then assayed with the Cell Proliferation Kit I (MTT test, Roche) according to the manufacturer’s instruction.
